# Home literacy practices for preschoolers: a scoping review

**DOI:** 10.1590/2317-1782/e20250148en

**Published:** 2026-07-10

**Authors:** Mirelly Danglês de Oliveira Ferreira, Brenda Albuquerque Adriano da Silva, Sarah Camila Ferreira de Oliveira Lima, João Victor Silva de Barros Lima, Karinna Veríssimo Meira Taveira, Cíntia Alves Salgado-Azoni

**Affiliations:** 1 Universidade Federal do Rio Grande do Norte – UFRN - Natal (RN), Brasil.; 2 Programa Associado de Pós-graduação em Fonoaudiologia, Universidade Federal do Rio Grande do Norte – UFRN - Natal (RN), Brasil.; 3 Instituto Santos Dumont – ISD - Macaíba (RN), Brasil.; 4 Programa de Pós-graduação em Psicologia, Universidade Federal do Rio Grande do Norte – UFRN - Natal (RN), Brasil.

**Keywords:** Preschool, Parents, Language, Learning, Early Intervention Educational

## Abstract

**Purpose:**

To map studies describing family literacy practices carried out with pre-school children.

**Research strategies:**

Consultation of Lilacs, EMBASE, PubMed, Scopus, LiVIVO and gray literature databases. Registration on the Open Science Framework (OSF) platform. Search strategies: “Child, Preschool”, “home literacy”, “Early Intervention Educational” and “Family”.

**Selection criteria:**

The acronym PCC was used: Population (Studies with preschoolers); Concept (family literacy practices); Context (family environment).

**Data analysis:**

Two authors carried out the identification and extraction of data: authors, year of publication, population, objective and the family literacy practices carried out with preschoolers.

**Results:**

For the final synthesis of the scoping review, 29 studies were included. Informal practices appeared most frequently.

**Conclusion**

The informal practice of reading books was the most described, followed by shared reading and teaching writing.

## INTRODUCTION

Family literacy is a set of resources and practices that a child experiences with their parents or caregivers. They encompass formal activities to teach them to read and write (e.g., recognize the alphabet, read and write words, and identify phonemes) and informal activities (e.g., experiences lived with the family that promote future learning, namely: interaction, outdoor play, various games, and music)^(1,2)^.

These practices are fundamental to strengthening family bonds and stimulating activities that help them prepare, from an early age, for the initial years of childhood education^(1-3)^.

Early childhood education includes nursery school and preschool. In most cases, it represents the first separation of children from their caregivers to incorporate them into structured socialization^(3,4)^.

Considering this process, preschool is the period that precedes literacy, divided into two axes: nursery school, which covers the age range from 0 to 3 years and 11 months, and preschool, which includes children from 4 to 5 years and 11 months^(4-6)^.

The learning rights stated in the Brazilian National Common Curriculum Base for preschoolers in early childhood education include spending time together, playing, participating, exploring, expressing, and knowing themselves. The concept that links education and care during this period has been gradually consolidated, understanding caregivers as inseparable from the educational process^(4)^.

Considering sociocultural and socioeconomic differences, children begin preschool with different academic skills, and one of the key factors influencing these differences is the family environment. Thus, daycare centers and preschools should learn about children’s experiences at home and in the community and articulate them with the pedagogical approach^(7)^. A stimulating family environment can help children develop cognitive and linguistic skills based on brain plasticity^(6,7)^.

Caregivers’ participation has significant and lasting effects on preschoolers’ early learning, not only on the initial academic results of preschool but also on the development of reading and writing when children begin to learn them^(8)^.

The quality of the family literacy environment may be associated with socioeconomic and educational indicators. However, research has indicated differences between sexes, favoring female children in home literacy environments, especially in book reading and formal literacy activities^(9)^.

Brazil established the Family Literacy Program in 2020, which recognizes the family as a key player in children’s educational success. The program includes guidelines for "fostering and promoting scientific research on family literacy and its impact on numeracy and literacy acquisition"^(1)^. The World Health Organization (WHO) has also cited concern, encouragement, and promotion of literacy practices, with recommendations for children under 5 years old, encouraging reading and storytelling with caregivers as alternatives to screen time to promote children’s health and development^(10)^.

The 2022 Programme for International Student Assessment (PISA) report highlighted the importance of recognizing the family as an essential actor in educational development and strengthening the partnership between school and family, aiming to involve parents in their children's learning process^(11)^. The document points out that students with better academic performance frequently report their families’ active participation in their learning activities^(11)^. Regarding reading results, student performance decreased by 10 points, which further emphasizes the need to promote and encourage family practices that contribute to learning in the early stages of development^(11)^.

Besides the assumptions mentioned above, studies indicate that improving education may be associated with reducing socioeconomic inequalities and that the lack of investment in education can have short- and long-term consequences for children and society. Hence, it is paramount to develop and implement measures that emphasize the importance of education, knowledge, and research^(12)^.

Thus, understanding the importance of the family environment for learning and the positive impacts that promoting family literacy can bring to society and the scientific community – a topic that is little discussed and researched in Brazil –, this study aimed to map, through a scoping review, the studies that describe family literacy practices with preschoolers.

## METHODS

This scoping review was registered in the Open Science Framework (OSF)^(13)^, developed according to the methodology of the Joanna Briggs Institute Reviewers’ Manual^(12)^, and reported according to the guidelines of the Preferred Reporting Items for Systematic Reviews and Meta-Analyses – Extension for Scoping Reviews (PRISMA-ScR)^(14)^.

### Eligibility criteria

The PCC acronym was used to determine the eligibility of articles to be included in this study:

Population (P): Studies with preschoolers (0 to 5 years and 11 months).
Concept (C): Family literacy practices.
Context (C): Family environment.

### Inclusion and exclusion criteria

The review included observational and experimental studies that described family literacy practices (i.e., activities with preschoolers [aged 0 to 5 years and 11 months] in the family environment). It excluded secondary studies, books, case reports, case series, letters, opinion articles, technical articles, and guidelines.

### Research sources and search strategy

Five databases were searched electronically: EMBASE, LIVIVO, Latin American and Caribbean Health Sciences Literature (LILACS), National Library of Medicine (PubMed), and Scopus. Additional literature sources, such as the Digital Library of Theses and Dissertations, were also searched. Keywords from Medical Subject Headings (MeSH) and Health Sciences Descriptors (DeCS), along with the Boolean operators AND and OR, were used to develop search strategies and conduct database searches. All sources were searched on March 2, 2024, and updated in March 2025.

### Selection

The Rayyan web application^(15)^ was used to manage and document the review process. Next, duplicate articles were excluded, and two authors (MDOF and BA) began the blind, independent review. Articles were first selected by analyzing titles and abstracts, and then two authors (MDOF and BA) read the full articles.

In both phases, articles not meeting the eligibility criteria were excluded. Disagreements during the independent reviews were discussed among the reviewers; if they persisted, the third author (SCFOL) reviewed the articles and applied her majority opinion.

### Data extraction and storage

Two authors identified and extracted data, which included authors, year of publication, population, objective, and family literacy practices with preschoolers.

### Analysis and presentation of results

The data were presented in figures and tables.

## RESULTS

### Selection of primary studies

**Figure 1:**
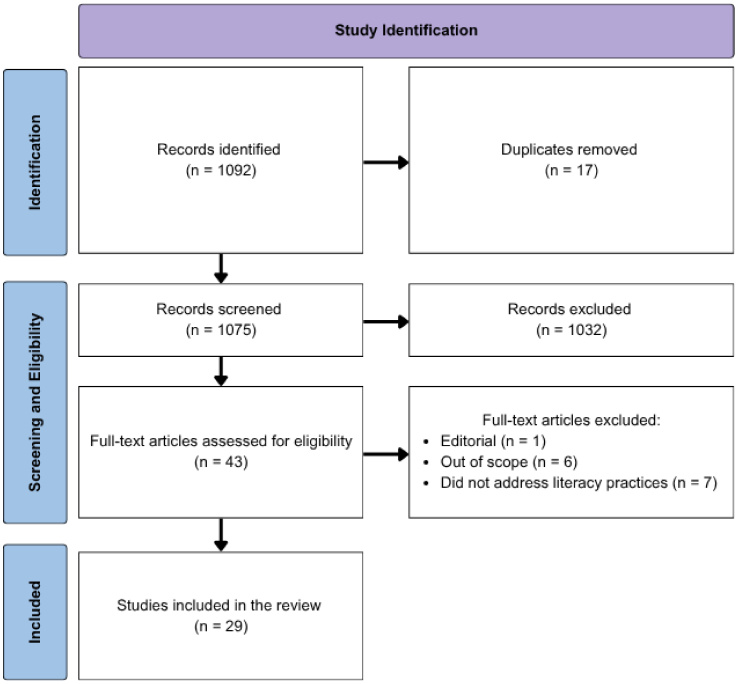
Identification of the studies

The database search strategy retrieved 1,092 references, totaling 1,075 after removing duplicates. The title and abstract reading phase excluded 1,032 articles that did not meet the eligibility criteria. Hence, 43 studies were selected for full-text reading, which excluded 14 studies, resulting in 29 studies included for the final synthesis of the scoping review (Figure 1).

### Characteristics of the studies

The included studies were published between 2001 and 2023 in various journals. All articles were published in English and conducted in different countries, including the United States (75.86%)^(16-37)^, Slovenia (3.44%)^(38)^, Chile (3.44%)^(39)^, Greece (3.44%)^(40)^, Australia (10.34%)^(41-43)^, and China (3.44%)^(44)^ (Figure 1).

The participants’ ages ranged from 9 months to 5 years and 11 months. All studies included both sexes. Sample sizes ranged from 20 to 11,173 participants. Only observational and experimental studies were selected.

All 29 studies cited family literacy practices; of these, 16 (55.17%) described formal and informal practices, 11 (37.96%) cited informal practices, and two (6.8%) reported only formal practices.

Informal activities appeared more often among family literacy practices. Twenty (68.96%) studies described the informal practice of reading books, followed by shared reading, cited in 10 (34.48%) studies, and teaching to write, mentioned in 10 (34.48%) studies.

Regarding informal practices, three (10.34%) studies cited storytelling as an activity, four (13.79%) articles mentioned singing songs, two (6.89%) studies cited playing, 10 (34.48%) cited shared reading, 20 (68.96%) mentioned reading to children, two (6.88%) reported using games with letters and numbers and talking to the child about how their day was, and seven (24.13%) cited coloring/drawing, reading before bed, teaching colors and shapes, and going to the library^(16,17,19-21,27,30,33,35,38,43)^ (Table 1).

**Table 1:** Summary of general descriptive characteristics of the included studies (n=29)

**Author**	**Participants**	**Type of study**	**Methods**	**Objectives**	**Home literacy practices**
Haney and Hill^(43)^	Preschoolers and their parents (n=47).	Cross-sectional, quantitative and qualitative study.	The Question from Home literacy Survey was administered to the parents of preschool children. The children were assessed using the Kaufman Survey of Early Academic and Language Skills (K-SEALS) and the Test. of Early Reading Ability—Third Edition (TERA-3).	To analyze how the family environment impacts emergent literacy.	Formal practices: Names and sounds of letters, writing words, reading words, or reading books. Informal practices: not mentioned
Boudreau^(23)^	Preschoolers and their parents (n=37).	Quantitative and qualitative cross-sectional study.	The Early Literacy Parent Questionnaire, consisting of 31 items, was administered to the parents of preschool children. In addition, the families answered open-ended questions about the literacy environment. The children were assessed on their rhyming skills, knowledge of printed elements in their surroundings, such as signs, brands, letter names and sounds, and storytelling.	To analyze the relationship between parents' knowledge of literacy and children's performance on early skills assessments.	Formal practices: Producing rhymes, writing, teaching letter names, letter sounds, phonological awareness, and the alphabet. Informal practices: storytelling skills and interactions with books.
Jay and Rohl^(41)^	Parents of preschoolers (n=9).	Longitudinal and quantitative experimental study.	An interview was conducted with the parents to investigate the home literacy environment. Following this, the families participated in an intervention program (workshops) with themes related to the development of reading and writing. Finally, they completed a self-assessment of the changes in the environment after the intervention.	To analyze an intervention program for parents to raise awareness about the literacy environment.	Informal practices: Reading books at bedtime, listening to recorded stories, going to the local library, storytelling, playing with alphabet puzzles, creating stories for children, reading newspapers, watching children's programs, going to the movies and the zoo, having picnics together. Formal practices: reading assignments such as homework, reading school books, discussing literacy, literacy-related activities.
Kim^(24)^	Preschoolers and their parents (n=215).	Longitudinal study.	The questionnaire on home literacy practices was administered to the parents.The children were assessed on vocabulary, knowledge of letter names, phonological awareness, word reading, pseudoword reading, and spelling.	To investigate the relationship between literacy practices and emerging literacy skills (vocabulary and phonological awareness) and conventional literacy skills (word reading, pseudoword reading, and spelling).	Formal practices: Teaching the alphabet and helping with homework. Informal practices: having children's books; shared reading, reading to children, visiting the library or bookstore.
Hammer et al.^(29)^	Preschoolers and their mothers (n=81).	Cross-sectional, descriptive study.	The Parental Modernity Scale and Rank Order of Parental Values ​​questionnaires were administered to the mothers of the children, investigating the beliefs, values, and practices used at home.	To analyze the language and literacy development of bilingual children.	Formal practices: writing letters, the alphabet, letter sounds, counting, teaching numbers, teaching writing, teaching shapes. Informal practices: Reading a book, teaching colors, coloring with the child, reading the Bible, reading the newspaper, reading a magazine, reading church newsletters, borrowing a book from the library, making a shopping list.
Li et al.^(37)^	Preschoolers (n=160)	Longitudinal study.	The Preschool and Primary Chinese Literacy Scale was administered to the children at the beginning of the study and again after three years. Parents answered a structured questionnaire about the frequency and type of literacy practices.	To investigate the effects of formal literacy activities on children's subsequent literacy outcomes.	Informal practices: Reading books and shared reading. Formal practices: teaching how to write.
Perry et al.^(28)^	Preschoolers and their mothers (n=13)	Longitudinal intervention study.	A structured questionnaire was administered to parents to assess reading and writing activities at home, use of reading materials, parental beliefs about literacy, and parental involvement in children's education. The questionnaire was administered at the beginning of the study and again two years later.	To examine how Latino immigrant families incorporate interactive, school-based literacy activities into their homes.	Informal practices: Literacy games and reading books. Formal practices: teaching colors, teaching body parts, associating the written word with oral language, reading written words aloud from books or games.
Hood et al.^(42)^	Preschoolers (n=143).	Longitudinal study.	The Home Literacy Environment Questionnaire, which assessed shared reading practices between parents and children, was administered to the parents, and the Children's Title Recognition Test was administered to the children.	To examine the practices carried out in the family environment and their relationship with emergent literacy.	Formal practices: Teaching the alphabet, teaching how to write one's name, teaching how to read.Informal practices: not mentioned.
Burgess^(22)^	Preschoolers and their parents (n=262).	Cross-sectional study.	A questionnaire developed by the researcher, containing questions about the home literacy environment, was administered to the parents.	Understanding the home literacy environment.	Informal practices: Reading children's books, reading magnetic letters, rhyming games.
Hindman and Morrison^(21)^	Preschoolers and their parents (n=229).	Cross-sectional study.	Parents and children were filmed/observed while performing a joint writing activity: writing an invitation to a birthday party. The task took place during a 1-hour home visit. Parents completed a questionnaire about literacy practices.	To analyze the development of literacy and literacy practices carried out during the spring.	Formal practices: Teaching letter sounds, teaching letter names, teaching how to read words, encouraging the child to write, doing activities related to mathematics. Informal practices: playing number games with children, supporting autonomy, encouraging the child to explore and question things, spending time with the child.
Skwarchuk et al.^(16)^	Preschoolers and their parents (n=279).	Cross-sectional study.	A questionnaire about literacy and numeracy was administered to the parents. The children answered tests that assessed numerical and reading knowledge.	Examine parents' academic expectations, literacy and numeracy attitudes, and literacy practices before their children start school.	Formal practices: Reading words, pointing to words/letters, recognizing written letters during reading, writing words, identifying words through signs (stop, continue), teaching letter sounds, presenting words and their meanings. Informal practices: singing songs that involve the alphabet, encouraging rhymes, asking questions after shared reading, visiting the library.
Förster and Rojas-Barahona^(39)^	Preschoolers and their parents (n=240).	Cross-sectional study.	A questionnaire about socioeconomic aspects and home literacy was administered to the parents. The children were assessed using the Assessment Battery for Children K-ABC.	To analyze the impact of literacy practices, sociodemographic variables, and attendance at a formal education program prior to the development of literacy skills.	Informal practices: Reading, singing songs, telling or talking about stories, playing games with letters and numbers, word games, reading signs or notices, reading magazines, talking about things that happened during the day, talking about television programs. Formal practices: writing letters and speeches.
Schick and Melzi^(30)^	Preschoolers and their parents (n=127).	Longitudinal study.	A questionnaire was administered to families regarding how often caregivers shared books with children, as well as the frequency of printed materials at home. The children were assessed for language skills.	To analyze home literacy practices at the beginning of the school year and the language, literacy, and socio-emotional school readiness outcomes of children at the end of the preschool year.	Informal practices: Reading books, shared reading, caregiver narrative creation, dialogue with the child, pointing out words on food labels and street signs, discussing recipes while cooking.Formal practices: asking children to write their name or other words.
Sawyer et al.^(31)^	Preschoolers and their mothers (n=20).	Cross-sectional study.	A semi-structured interview was conducted with the mothers regarding their home literacy practices, beliefs about how children learn to read, write, and use language, and factors that influence these practices and beliefs.	To analyze the environment of home literacy practices, what beliefs mothers have about how children learn to read, and what factors influenced the mothers' beliefs and practices.	Informal practices: Going to the library, reading books, coloring/drawing, engaging with books. Formal practices: Not mentioned.
Bingham et al.^(20)^	Preschoolers and their parents (n=181).	Exploratory study.	Self-report questionnaires were administered to parents to indicate the extent to which they agreed with statements representative of permissive and authoritarian parenting styles, and a home literacy form divided into formal and informal activities was completed. The children's oral language was assessed using the following tests: Peabody Picture Vocabulary Test – Fourth Edition (PPVT-4) and Test of Preschool Early Literacy (TOPEL).	To analyze parenting styles and literacy practices and their correlations with oral language skills.	Informal practices: Shared reading of books, reading books, going to the library, reading illustrated books.
Gonzalez et al.^(32)^	Preschoolers and their parents (n=252).	Randomized clinical trial.	A questionnaire was administered to the mothers regarding the socioeconomic context, maternal education, literacy environment, maternal reading beliefs, and frequency of shared reading. The children were assessed for vocabulary.	To analyze whether the sociodemographic characteristics of parents are associated with the beliefs and attitudes that parents have about the language and literacy development of their children.	Informal practices: Reading books.
van Bysterveldt and Westerveld^(45)^	Preschoolers and their parents (n=77).	Cross-sectional study.	Two questionnaires were administered: a sociodemographic questionnaire and the Zarit Burden Interview (12-item version).	Analyze emerging literacy and cognitive skills.	Informal practices: Reading books, having books at home, shared reading, rhyming games. Formal practices: reading words, letter names, writing words, naming words.
Marjanovič-Umek et al.^(38)^	Preschoolers and their mothers (n=20).	Observational study.	The Scale for Observing Shared Reading was used to assess the quality of shared reading, the Frog Goes to Dinner quiz was used to evaluate the child's narrative, and a questionnaire about the home literacy environment was administered.	To analyze the relationships between the quality of shared mother-child reading, the child's storytelling ability, and the literacy environment at home.	Informal practices: Imitates the child's voice or onomatopoeia, reads books, shared reading, talks to the child about the illustrations, answers the child's questions, praises the child; Connects the story content to the child's daily life and real experiences; Describes feelings and desires; Describes mental states; Uses metaphors; Asks questions about cause and effect relationships; Asks questions that involve metacognitive processes; Joint attention with a child. Formal practices: explains the characteristics of words, asks the child to explain the meaning of words.
Puranik et al.^(18)^	Preschoolers and their parents (n=151).	Cross-sectional study.	A questionnaire about home literacy practices was administered to the parents, and an assessment of the children's writing skills was conducted.	To analyze what types of writing-related activities parents carry out with their preschool-aged children and whether literacy practices at home contribute to the children's initial writing skills.	Formal practices: Teaching letters, writing letters, writing one's name, writing activities together, writing notes or birthday cards, writing names/words. Informal practices: not mentioned.
Roopnarine and Dede Yildirim^(19)^	Preschoolers and their parents (n=11,173)	Cross-sectional study.	A questionnaire was administered to parents regarding engagement in cognitive activities, literacy resources at home, and children's literacy skills.	To analyze the involvement of mothers and fathers in play, reading books, and storytelling activities.	Informal practices: Homemade toys, store-bought toys, reading books, telling stories, and playing. Formal practices: identifying letters, reading words.
Riser et al.^(17)^	Preschoolers and their parents (n=10,400)	Cross-sectional study.	The involvement of parents in home literacy activities was observed, and the children were assessed in relation to their academic skills.	Secondary data analysis included interviews and questionnaires with parents, birth certificate records, home visits, teachers, classroom observations, and direct assessments of the children.	Informal practices: Shared reading, storytelling, and singing songs. Formal practices: not mentioned.
Myrtil et al.^(25)^	Preschoolers and their parents (n=466)	Cross-sectional study.	A questionnaire about home literacy practices was administered to the parents, and the children's oral language was assessed.	To examine the interrelationship of the home literacy environment of low-income rural families (interactions between parents and children, child's interests, library use and access to books) and to determine the extent to which caregiver and child characteristics predict these dimensions.	Informal practices: Reading books, singing and reciting rhymes, telling stories, drawing, going to the library. Formal practices: writing letters.
Lin et al.^(33)^	Preschoolers and their parents (n=262).	Correlational study.	Questionnaires were administered regarding literacy practices, communication between parents and educators, and demographic variables.	To examine the relationship between parents' perceptions of communication between parents and educators and children's home learning environments.	Informal practices: Reading books containing numbers, reading books. Formal practices: naming written letters, identifying letter sounds.
Eutsler and Trotter^(35)^	Preschoolers and their parents (n=37)	This study involves multiple cases.	A questionnaire about literacy practices was administered to the parents. Shared reading sessions (parents and child) were conducted, with two sessions using printed texts and two using digital texts. Behavioral observation of the children was also carried out.	To analyze children's home literacy practices and reading preferences in a classroom setting within the school environment.	Informal practices: Reading printed or digital books, imaginative games, technological devices.
Chen et al.^(26)^	Preschoolers and their parents (n=4,907).	Cross-sectional study.	A questionnaire about literacy practices and demographic variables was administered to the parents.	To investigate how different devices (including TV, tablet, computer and paper books) can channel parental effectiveness (or lack thereof) toward home literacy practices.	Informal practices: Reading books and storytelling.
Tsirmpa et al.^(40)^	Preschoolers and their parents (n=147).	Cross-sectional study.	Parents responded to semi-structured interviews about home literacy beliefs and practices.	To analyze beliefs about literacy and the development of literacy.	Informal practices: Reading books, naming colors. Formal practices: pointing to letters or numbers.
Dulin et al.^(27)^	Preschoolers and their mothers (n=13)	Longitudinal study	A questionnaire about literacy practices was administered to the parents. The children were assessed using the MacArthur-Bates test.	To characterize the home literacy environments of children with Down syndrome and to examine whether the richness of the literacy environment, the child's involvement during shared reading activities of storybooks, the quality of the shared reading activity of storybooks between caregiver and child, and exposure to language in the home environment simultaneously predicted the child's receptive vocabulary.	Informal practices: Shared reading, reading books, storytelling, naming pictures in books. Formal practices: Not mentioned.
DesJardin et al.^(36)^	Preschoolers and their parents (n=36).	Cross-sectional study.	A questionnaire about literacy practices was administered to the parents. The children were assessed in relation to oral language and shared reading interactions.	To investigate the quantity (e.g., frequency of shared reading, child's enjoyment) and quality (parent and child behaviors) of shared book reading among preschool children with and without hearing loss.	Informal practice: Shared reading. Formal practice: not cited.

The following practices were cited in 1 (3.44%) study^(16,17,19-21,27,30,33,35,38,43)^: talking to the child about the book's illustrations, answering the child's questions, complimenting them, connecting reading content to daily life activities, making toys, watching videos together, playing with puzzles, going to the movies, going to the zoo, having a picnic, and reading the Bible, magazines, and newspapers (Table 1).

Regarding formal practices, 10 (34.48%) studies cited writing words, two (6.88%) cited teaching to read, teaching the alphabet, doing school activities, and teaching the letters’ names, five (17.20%) studies cited rhyme production, and one (3.44%) study mentioned teaching to write their names and identifying graphemes and phonemes^(16,18,21,23,42)^ (Table 1).

The literacy practices cited in these studies were carried out by parents, with the mother figure predominating in both formal and informal activities.

## DISCUSSION

This scoping review compiled data regarding family literacy practices with preschoolers, which the studies described here classify as formal and informal. Formal practices are related to teaching the letters’ names and sounds, reading words, and writing. These activities are associated with alphabet recognition in preschool, developing predictive skills for written language^(16,17)^.

Informal practices refer to activities that stimulate children's development and will later be associated with the skills necessary for formal learning to read and write. The reviewed studies described practices such as shared reading, storytelling, visits to the library, singing, making their own toys, drawing, and watching videos together^(16,20,21,23,38)^.

There is a relationship between informal practices and oral language skills, as exemplified by the studies of Dulin et al.^(27)^, Bingham et al.^(20)^, Puranik et al.^(18)^, Marjanovič-Umek et al.^(38)^, Skwarchuk et al.^(16)^, Hindman and Morrison^(21)^ and Kim^(24)^, who investigated informal practices and their impacts on language^(16,17,20,21,27,38)^. The articles presented practices such as reading books, offering books to children, shared reading, and activities with music, and related these activities to better performance in cognitive assessments and impacts on vocabulary, phonological awareness, and word reading^(16,20,21,38)^.

The study by Riser et al.^(17)^ with 10,400 preschoolers of indigenous descent emphasized the association of positive results on a cognitive scale with informal practices from the perspective of activities related to their own culture, through storytelling and songs^(17)^. Literacy practices were also associated with income and maternal education level.

Informal practices were also described in the study by Roopnarine and Dede Yildirim^(19)^, which aimed to compare maternal and paternal participation in the family environment. Only one of the five countries surveyed in this study had more active participation of fathers in free play, storytelling, and toy making; in the other countries, mothers were much more likely to engage in these practices. Moreover, storytelling and play with mothers were significantly and positively associated with letter identification^(19)^.

The involvement of mothers in literacy practices and their consequences to children's development was explored in the study by Bingham et al.^(20)^, which verified the quality of shared reading between mother and child. Moreover, the studies by Hammer et al.^(29)^, Tsirmpa et al.^(40)^, Burgess^(22)^ and Dulin et al.^(27)^ found greater involvement of the maternal figure in doing activities^(27,29,40)^. This may be associated with the culture of mothers caring for children – when we examine studies that portray the primary caregiver, the maternal figure is highlighted as more active in this care^(44,46,47)^.

Regarding formal practices, studies by Puranik et al.^(18)^, Skwarchuk et al.^(16)^, Hindman and Morrison^(21)^, Hood et al.^(42)^ and Kim^(24)^ correlated activities involving writing with their consequences for incipient learning to write, as well as the impacts on oral language and reading skills^(16,18,21,23,42)^. The research by Puranik et al.^(18)^ also emphasized the correlations between parental education and children's letter writing, spelling, and spontaneous writing skills.

Kim^(24)^ study examined formal and informal practices and their consequences to learning to read words and pseudowords. The comparison between preschoolers doing formal activities, such as teaching the letters’ names, found that those exposed to reading performed better in reading^(24)^.

In Perry et al.^(28)^ research, which aimed to verify preferences between formal and informal activities, parents reported preferring informal practices focused on play, involving board games, drawing, and reading books in a playful way with variations in vocal intonations.

Despite the preference for informal activities, parents reported supporting the development of their children's knowledge of numbers and vocabulary through activities such as repetitive counting and practice of letter and sound associations^(28)^. Furthermore, parents identified literacy activities as tools to convey a moral message or guide children toward good behavior^(28)^.

Tsirmpa et al.^(40)^ research observed parents' beliefs regarding the types of practices that should be carried out with preschoolers. It found that more conventional parents believed that children should be familiar with letters and numbers and be able to recognize and write some of them. In contrast, parents who favored facilitative education believed that fine motor skills, visual perception, and hand-eye coordination should be worked on, as well as oral language, to promote the learning of written language.

Regarding the education level, studies by Riser et al.^(17)^, Puranik et al.^(18)^ and Skwarchuk et al.^(16)^ observed an association between literacy practices, better performance in language assessments, and the parents’ education level^(16-18,22,40)^. They also found that parents with a higher education level considered the family as a primary factor in preschoolers’ teaching-learning process, while parents with a lower education level believed that the school is primarily responsible for teaching children^(40)^.

In contrast to the studies cited above, the research by Förster and Rojas-Barahona^(39)^ and Burgess^(22)^ found no difference between education levels and activities involving reading^(22,39)^. On the other hand, socioeconomic income was associated with a greater availability of resources such as books, letter games, and toys^(25,26)^.

Furthermore, all studies included in the review cited the informal practice of reading books and the impacts of family literacy practices on children's development. When compared with findings in the literature, several studies emphasize family participation as essential for educational success and linguistic and social development; thus, these data are in agreement^(48,49)^.

Moreover, 27 of the 29 studies included in the review were carried out in developed countries^(16-37)^. This data may be related to these countries’ greater concern with children’s development and education, since children out of school or who drop out of school are among the most important social problems in the world, especially in low- and middle-income countries.

According to Article 26 of the Universal Declaration of Human Rights, countries need to provide everyone with access to free education and invest significant resources in educational facilities. However, this review found only one study conducted in a developing country, which may be related to the school dropout rate mentioned in the study above and associated with educational indicators in developing countries.

Thus, the continuation of research aimed at exploring aspects of family literacy is paramount. Moreover, experimental studies that observe the results of literacy activities with socially vulnerable families can contribute to education, reduce school dropout rates, and improve educational indicators in developing countries.

Regarding the limitations of the present study, it is possible to highlight methodological aspects of the articles found, such as the absence of a control group in the intervention studies and the random distribution of participants. As for the observational studies, the use of semi-structured interviews with open-ended questions to investigate the types of practices may have compromised the results compared to those that used validated protocols.

## CONCLUSION

The mapping of practices with preschoolers shows the predominance of informal practices. The most cited activity was reading books, followed by shared reading. Furthermore, playing together, making their own toys, drawing, coloring, singing, listening to music, and going on outings were activities reported by caregivers. Teaching to write was the most described formal practice. Finally, there is a consensus on the importance of the family environment in children's learning and the relationship between family literacy practices and the development of oral language and, subsequently, written language.

## Material Suplementar

Este artigo acompanha material suplementar.

Legenda S1 Apêndice A. Estratégia De Pesquisa Em Banco De Dados

Legenda S2 Apêndice B. Artigos Excluídos

Este material está disponível como parte da versão online do artigo na página https://doi.org/ DOI: 10.1590/2317-1782/e20250148en
