# Supplementary material for: Home literacy practices for preschoolers: a scoping review
Source: Codas. 2026 Jul 10;38(4):e20250148. doi: 10.1590/2317-1782/e20250148en (PMC13359255; doi:10.1590/2317-1782/e20250148en)
Supplement: Legenda S1 [file codas-38-4-e20250148-suppl01-en.pdf]

Appendix A. Database Search Strategy

Database:	Search:
PubMed	((((("Child, Preschool"[Mesh] OR "Infant"[Mesh] OR "Infant, Newborn"[Mesh] OR "Preschool Child" OR "Preschool Children" OR Infant OR Infants OR child OR children OR kid OR kids OR preschooler OR preschoolers OR "preschool children's" OR todler OR "Newborn Infant" OR "Newborn Infants" OR Newborns OR Newborn OR Neonate OR Neonates)) AND (("home literacy" OR "home literacy environment" OR "Home Literacy Learning" OR "Home Environment and Learning" OR "family learning environment")))) AND (("Early Intervention Educational" OR "Early Intervention" OR intervention)))) AND (("Family"[Mesh] OR "Fathers"[Mesh] OR "Parents"[Mesh] OR "Mothers"[Mesh] OR "Parents" OR "Parent" OR Parents' "Parenthood Status" OR "Step-Parents" OR "Step Parents" OR "Step-Parent" OR "Stepparent" OR "Stepparents" OR "Parental Age" OR "Parental Ages" OR "Mothers' Clubs" OR "Mother Clubs" OR Mother OR Father OR Family OR Fathers OR Mothers))
SCOPUS	("Criança Pré-Escolar" OR "Crianças Pré-Escolares" OR "Pré-Escolares" OR "Child, Preschool" OR "Preescolar" OR "Enfant d'âge préscolaire") AND ("Relações Pais-Filho" OR "Interação entre Pais e Filhos" OR "Interação Pais-Criança" OR "Interação Pais-Filho" OR "Interação Pais-Filhos" OR "Relações entre Pais e Filhos" OR "Parent-Child Relations" OR "Relaciones Padres-Hijo" OR "Relations parent-enfant") AND ("home literacy" OR "home literacy environment" OR "Home Literacy Learning" OR "Home Environment and Learning" OR "family learning environment") AND ("Educação de Intervenção Precoce" OR "Estimulação Precoce" OR "Intervenção Precoce" OR "Intervenção Precoce Educacional" OR "Intervenção Precoce na Escola" OR "Intervenção Precoce nas Escolas" OR "Intervenção Precoce, Educação" OR "Early Intervention, Educational" OR "Intervención Educativa Precoz")
Embase	('child, preschool'/exp OR 'child, preschool' OR (('child,'/exp OR child,) AND ('preschool'/exp OR preschool))) AND ('home literacy learning' OR (('home'/exp OR home) AND ('literacy'/exp OR literacy) AND ('learning'/exp OR learning))) AND ('parent'/exp OR parent)
LILACS	("Criança Pré-Escolar" OR "Crianças Pré-Escolares" OR "Pré-Escolares" OR "Child, Preschool") AND ("home literacy" OR "home literacy environment" OR "Home Literacy Learning" OR "Home Environment and Learning" OR "family learning environment") AND ("Educação de Intervenção Precoce" OR "Estimulação Precoce" OR "Intervenção Precoce" OR "Intervenção Precoce Educacional" OR "Intervenção Precoce na Escola" OR "Intervenção Precoce nas Escolas" OR "Intervenção Precoce, Educação" OR "Early Intervention, Educational")
LiVIVO	('child, preschool'/exp OR 'child, preschool' OR (('child,'/exp OR child,) AND ('preschool'/exp OR preschool))) AND ('home literacy learning' OR (('home'/exp OR home) AND ('literacy'/exp OR literacy) AND ('learning'/exp OR learning))) AND ('parent'/exp OR parent)

BDTD	('child, preschool'/exp OR 'child, preschool' OR (('child,'/exp OR child,) AND ('preschool'/exp OR preschool))) AND ('home literacy learning' OR (('home'/exp OR home) AND ('literacy'/exp OR literacy) AND ('learning'/exp OR learning))) AND ('parent'/exp OR parent)

Appendix B. Excluded Articles

Editorial: The impact of home and school environment on early literacy and mathematic skills	BONIFACCI, Paola; TOBIA, Valentina; INOUE, Tomohiro; MANOLITSIS, George. Editorial: the impact of home and school environment on early literacy and mathematic skills. Frontiers In Psychology, [S.L.], v. 14, n. 2, p. 1-34, 8 ago. 2023. Frontiers Media SA. http://dx.doi.org/10.3389/fpsyg.2023.1258391.	Editorial
Home and Clinical Literacy Practices for Children With Cleft Lip and Palate	POROD, Therese K.; GORMAN, Brenda K.. Home and Clinical Literacy Practices for Children With Cleft Lip and Palate. The Cleft Palate Craniofacial Journal, [S.L.], v. 57, n. 10, p. 1216-1229, 13 maio 2020. SAGE Publications. http://dx.doi.org/10.1177/1055665620924938.	Outside the age range
Home literacy environment profiles of children with language impairment: associations with caregiver- and child-specific factors.	TAMBYRAJA, Sherine R.; SCHMITT, Mary Beth; FARQUHARSON, Kelly; JUSTICE, Laura M.. Home literacy environment profiles of children with language impairment: associations with caregiver- and child-specific factors. **International Journal Of Language & Communication Disorders**, [S.L.], v. 52, n. 2, p. 238-249, 10 jul. 2016. Wiley. http://dx.doi.org/10.1111/1460-6984.12269.	Outside the age range
The influence of early home literacy activities and home resources for learning on fourth grade students' reading performance	CHIU, Chiahui. The influence of early home literacy activities and home resources for learning on fourth grade students' reading performance. **Bulletin Of Educational Psychology**, Taiwan, v. 52, n. 3, p. 685-706, ago. 2021.	Outside the age range
Home and school interventions aided at-risk students' literacy during Covid-19: a longitudinal	DUNN, Kristy; GEORGIOU, George K.; INOUE, Tomohiro; SAVAGE, Robert; PARRILA, Rauno. Home and school interventions aided at-risk students' literacy during Covid-19: a longitudinal analysis. **Reading And Writing**, [S.L.], v. 36, n. 2, p. 449-466, 14 nov. 2022. Springer Science and Business Media LLC. http://dx.doi.org/10.1007/s11145-022-10354-7.	Outside the age range
Home Literacy Environment and Early Literacy Development Across Languages Varying in Orthographic Consistency	INOUE, Tomohiro; MANOLITSIS, George; JONG, Peter F. de; LANDERL, Karin; PARRILA, Rauno; GEORGIOU, George K.. Home Literacy Environment and Early Literacy Development Across Languages Varying in Orthographic Consistency. **Frontiers In Psychology**, [S.L.], v. 11, n. 7, p. 1-11, 31 jul. 2020. Frontiers Media SA. http://dx.doi.org/10.3389/fpsyg.2020.01923.	Outside the age range
Impact of home literacy environment on literacy	WANG, Qianqian; MA, Minjie; HUANG, Yan; WANG, Xichen; WANG, Tingzhao. Impact of home literacy environment on literacy	Outside the age range

development of children with hearing loss: A mediation model	development of children with hearing loss: a mediation model. **Frontiers In Psychology**, [S.L.], v. 13, n. 1, p. 1-10, 14 out. 2022. Frontiers Media SA. http://dx.doi.org/10.3389/fpsyg.2022.895342.	
Identifying the preschool home learning experiences that predict early number skills: Evidence from a longitudinal study	SOTO-CALVO, Elena; SIMMONS, Fiona R.; ADAMS, Anne-Marie; FRANCIS, Hannah N.; PATEL, Hannah; GIOFRÈ, David. Identifying the preschool home learning experiences that predict early number skills: evidence from a longitudinal study. **Early Childhood Research Quarterly**, [S.L.], v. 53, n. 1, p. 314-328, 2020. Elsevier BV. http://dx.doi.org/10.1016/j.ecresq.2020.04.004.	He did not mention the types of family literacy practices.
Long-term effects of the home literacy environment on reading development: familial risk for dyslexia as a moderator.	TORPPA, Minna; VASALAMPI, Kati; EKLUND, Kenneth; NIEMI, Pekka. Long-term effects of the home literacy environment on reading development: familial risk for dyslexia as a moderator. **Journal Of Experimental Child Psychology**, [S.L.], v. 215, p. 105314, mar. 2022. Elsevier BV. http://dx.doi.org/10.1016/j.jecp.2021.105314..	He did not mention the types of family literacy practices.
Increasing Caregivers' Adherence to an Early-Literacy Intervention Improves the Print Knowledge of Children with Language Impairment	JUSTICE, Laura M.; CHEN, Jing; TAMBYRAJA, Sherine; LOGAN, Jessica. Increasing Caregivers' Adherence to an Early-Literacy Intervention Improves the Print Knowledge of Children with Language Impairment. **Journal Of Autism And Developmental Disorders**, [S.L.], v. 48, n. 12, p. 4179-4192, 3 jul. 2018. Springer Science and Business Media LLC. http://dx.doi.org/10.1007/s10803-018-3646-2.	He did not mention the types of family literacy practices.
Laying a firm foundation: Embedding evidence-based emergent literacy practices into early intervention and preschool environments	TERRELL, Pamela; WATSON, Maggie. Laying a Firm Foundation: embedding evidence-based emergent literacy practices into early intervention and preschool environments. **Language, Speech, And Hearing Services In Schools**, [S.L.], v. 49, n. 2, p. 148-164, 5 abr. 2018. American Speech Language Hearing Association. http://dx.doi.org/10.1044/2017_lshss-17-0053.	He did not mention the types of family literacy practices.
Dialogic reading with attention-deficit-hyperactivity disorder (ADHD) kindergarteners: Does reading with parents or siblings enhance their language development?	DONG, Yang; CHOW, Bonnie Wing-Yin; MO, Jianhong; ZHENG, Hao-Yuan. Dialogic reading with attention-deficit-hyperactivity disorder (ADHD) kindergarteners: does reading with parents or siblings enhance their language development?. **Developmental Psychology**, [S.L.], v. 59, n. 5, p. 862-873, maio 2023. American Psychological Association (APA). http://dx.doi.org/10.1037/dev0001466.	He did not mention the types of family literacy practices.
Dialogic reading and morphology training in Chinese children: effects on language and literacy.	CHOW, Bonnie Wing-Yin. Dialogic Reading and Morphology Training in Chinese Children: Effects on Language and Literacy. **Developmental Psychology**, [*s. l*], v. 44, n. 1, p. 233-244, abr. 2008.	He did not mention the types of family literacy practices.
Associations between home literacy environment, brain white matter integrity and cognitive abilities in preschool‐age children.	HUTTON, John S.; DUDLEY, Jonathan; HOROWITZ‐KRAUS, Tzipi; DEWITT, Tom; HOLLAND, Scott K.. Associations between home literacy environment, brain white matter integrity and cognitive abilities in preschool‐age children. **Acta Paediatrica**, [S.L.], v. 109, n. 7, p. 1376-1386, 18 dez. 2019. Wiley. http://dx.doi.org/10.1111/apa.15124.	He did not mention the types of family literacy practices.
