# Supplementary material for: Home literacy practices for preschoolers: a scoping review
Source: Codas. 2026 Jul 10;38(4):e20250148. doi: 10.1590/2317-1782/e20250148en (PMC13359255; doi:10.1590/2317-1782/e20250148en)
Supplement: Translation

# Translation: Práticas de literacia familiar para pré-escolares: uma revisão de escopo

**DOI:** 10.1590/2317-1782/e20250148pt

**Authors:** Mirelly Danglês de Oliveira Ferreira, Brenda Albuquerque Adriano da Silva, Sarah Camila Ferreira de Oliveira Lima, João Victor Silva de Barros Lima, Karinna Veríssimo Meira Taveira, Cíntia Alves Salgado-Azoni

**Affiliations:** 1 Universidade Federal do Rio Grande do Norte – UFRN - Natal (RN), Brasil.; 2 Programa Associado de Pós-graduação em Fonoaudiologia, Universidade Federal do Rio Grande do Norte – UFRN - Natal (RN), Brasil.; 3 Instituto Santos Dumont – ISD - Macaíba (RN), Brasil.; 4 Programa de Pós-graduação em Psicologia, Universidade Federal do Rio Grande do Norte – UFRN - Natal (RN), Brasil.

**Keywords:** Pré-Escolar, Pais, Linguagem Infantil, Aprendizagem, Estimulação Precoce

## Abstract

**Objetivo:**

Mapear os estudos que descrevem as práticas de literacia familiar realizadas com crianças pré-escolares.

**Estratégia de pesquisa:**

Consulta nas bases de dados da Lilacs, EMBASE, PubMed, Scopus, LiVIVO e literatura cinzenta. Registro na plataforma Open Science Framework (OSF).

**Estratégia de pesquisa:**

“Child, Preschool”, “home literacy", “Early Intervention Educational” e "Family”.

**Critérios de seleção:**

Foi utilizado o acrônimo PCC: População (Estudos com pré-escolar); Conceito (práticas de literacia familiar); Contexto (ambiente familiar).

**Análise dos dados:**

Dois autores realizaram a identificação e extração dos dados: autores, ano de publicação, população, objetivo e as práticas de literacia familiar realizadas com os pré-escolares.

**Resultados:**

Para a síntese final da revisão de escopo foram incluídos 29 estudos. As práticas informais apareceram com mais frequência.

**Conclusão:**

A prática informal de leitura de livros foi a mais descrita, seguida da leitura compartilhada e o ensino da escrita.

## INTRODUÇÃO

A literacia familiar é um conjunto de recursos e práticas que a criança vivencia com seus pais ou cuidadores, dentre elas, podemos citar atividades formais, referentes ao ensino da leitura e da escrita, que envolvem reconhecimento do alfabeto, leitura e escrita de palavras e identificação dos fonemas, e informais, que envolvem experiências vivenciadas com a família que promovem aprendizagem futura, a saber: interação, brincadeiras ao ar livre, jogos diversos e músicas^(1,2)^.

Essas práticas são fundamentais para fortalecer o vínculo familiar e promover desde cedo o estímulo de atividades que contribuem para preparação aos anos iniciais da educação infantil^(1-3)^.

O processo da educação infantil, compreende a entrada na creche ou na pré-escola, e em sua maioria, configura a primeira separação das crianças dos seus cuidadores para se incorporarem a uma situação de socialização estruturada^(3,4)^.

Ao pensarmos nesse processo, a Educação Infantil é o período que antecede a alfabetização, e esta se divide em dois eixos: a creche que abrange a faixa etária de 0 a 3 anos e 11 meses, e a pré-escola, que compreende as crianças de 4 a 5 anos e 11 meses^(4-6)^.

No Brasil, os direitos de aprendizagem presentes na Base Nacional Comum Curricular, que devem acontecer no Ensino Infantil para os pré-escolares, estão o conviver, brincar, participar, explorar, expressar e conhecer-se. Nesse período, vem se consolidando a concepção que vincula educar e cuidar, entendendo o cuidador como um sujeito indissociável do processo educativo^(4)^.

Considerando diferenças socioculturais e socioeconômicas, as crianças iniciam a fase pré-escolar com capacidades acadêmicas diferentes, e um dos fatores-chave que influenciam essas diferenças é o ambiente familiar. Dessa forma, é proposto que as creches e pré-escolas, ao conhecer as vivências experienciadas pelas crianças no ambiente familiar e no contexto de sua comunidade, façam uma articulação com as propostas pedagógicas^(7)^. Isso porque, um ambiente familiar estimulador, pode favorecer o desenvolvimento de habilidades cognitivas e linguísticas a partir da plasticidade cerebral^(6,7)^.

A participação dos cuidadores na aprendizagem precoce dos pré-escolares tem efeitos significativos e duradouros, não apenas nos resultados acadêmicos iniciais da pré-escola, mas também no desenvolvimento da leitura e da escrita quando a criança inicia o processo de alfabetização^(8)^.

A qualidade do ambiente de literacia familiar pode estar associada a indicadores socioeconômicos e educacionais, mas algumas pesquisas indicaram que também existem diferenças de gênero, em que favorecem as crianças do gênero feminino nos ambientes de alfabetização doméstica, principalmente nas atividades que envolvem leitura de livros e atividades formais de alfabetização^(9)^.

No Brasil, em 2020, foi publicado o Programa de Literacia Familiar, que reconhece a família como ator fundamental para o sucesso educacional dos filhos. Além disso, o programa inclui diretrizes para o “[...] fomento e a promoção de pesquisa científica sobre literacia familiar e seu impacto na aquisição de numeracia e literacia”^(1)^. A preocupação, o incentivo e a promoção das práticas de literacia foram citadas também pela Organização Mundial da Saúde (OMS), que divulgou recomendações para crianças menores de 5 anos, incentivando a leitura e a narração de histórias com os cuidadores como alternativas ao tempo de tela para promover a saúde e o desenvolvimento infantil^(10)^.

O relatório do *Programme for International Student Assessmen*t (PISA) de 2022 destacou a importância de reconhecer a família como um ator essencial no desenvolvimento educacional e de fortalecer a parceria entre escola e família, visando envolver os pais no processo de aprendizagem dos filhos^(11)^. No documento, foi observado que os alunos com melhor desempenho acadêmico relatam frequentemente a participação ativa de suas famílias em suas atividades de aprendizagem^(11)^. Em relação aos resultados de leitura, houve uma diminuição de 10 pontos no desempenho dos estudantes, o que enfatiza ainda mais a necessidade de promover e incentivar práticas familiares que contribuam para a aprendizagem nas fases iniciais do desenvolvimento^(11)^.

Dessa forma, embasado nos pressupostos acima citados e, levando em consideração estudos que indicam que melhorar a educação pode estar associado à redução das desigualdades socioeconômicas, bem como que a falta de investimentos na educação podem provocar consequências a curto e longo prazo para a criança e para a sociedade, desenvolver e implementar medidas que enfatizem a importância da educação, do conhecimento e da pesquisa é primordial^(12)^.

Portanto, compreendendo a importância do ambiente familiar para aprendizagem, bem como os impactos positivos que a promoção da literacia familiar pode trazer para sociedade e para comunidade científica, uma vez que, se refere a um tema pouco discutido e pesquisado no Brasil, este estudo objetiva por meio de uma revisão de escopo, mapear os estudos que descrevem quais as práticas de literacia familiar realizadas com pré-escolares.

## MÉTODO

Esta revisão de escopo foi registrada na Open Science Framework (OSF)^(13)^ e desenvolvida conforme a metodologia do Joanna Briggs *Institute Reviewers’ Manual* ^(12)^, e relatada segundo o guia para relatório de revisão de escopo *Preferred Reporting Items for Systematic reviews and Meta-Analyses extension for Scoping Reviews* (PRISMA-ScR)^(14)^.

### Critérios de elegibilidade

Como forma de determinar a elegibilidade dos artigos a serem incluídos no presente estudo, foi utilizado o acrônimo PCC:

População (P): Estudos com pré-escolar (0 a 5 anos e 11 meses);
Conceito (C): Práticas de literacia familiar;
Contexto (C): Ambiente familiar.

### Critérios de inclusão e exclusão

Estudos observacionais e experimentais que descreveram as práticas de literacia familiar, ou seja, as atividades realizadas com as crianças no ambiente familiar para os pré-escolares (faixa etária de 0 a 5 anos e 11 meses de idade), foram incluídos. Os estudos secundários, livros, relatos de casos, séries de casos, cartas, artigos de opinião e artigos técnicos e diretrizes foram excluídos.

### Fontes de pesquisa e estratégia de busca

Cinco bases de dados foram pesquisadas na busca eletrônica: EMBASE, LIVIVO, Literatura Latino-Americana e do Caribe em Ciências da Saúde (Lilacs), National Library of Medicine (PubMed) e Scopus. Também foram pesquisadas fontes de estudos na literatura adicional, como Biblioteca Digital de Tese e Dissertações. Foram utilizadas as palavras-chave do Medical Subject Headings (MeSH) e Descritores em Ciências da Saúde (DeCS), em conjunto com os operadores booleanos *AND* e *OR*, para elaborar as estratégias de busca e realizar as pesquisas nas bases de dados. Todas as fontes foram pesquisadas em 02 de março de 2024 e atualizadas em março de 2025.

### Seleção

Foi usado para gerenciar e documentar o processo de revisão, o aplicativo da web Rayyan^(15)^. Em seguida, os artigos duplicados foram excluídos e deu-se início a análise cega e independente por dois autores (MDOF e BA). Os artigos foram selecionados primeiramente por meio da análise dos títulos e resumo e, em seguida, (MDOF e BA) fizeram a leitura dos artigos completos.

Em ambas as fases, quando os critérios de elegibilidade não foram contemplados, os artigos foram excluídos. As discordâncias durante as avaliações independentes foram discutidas entre os revisores e ao persistir, o terceiro autor (SCOFL) revisou os artigos e aplicou sua opinião majoritária.

### Extração e armazenamento dos dados

Dois autores realizaram a identificação e extração dos dados. Os dados extraídos foram: autores, ano de publicação, população, objetivo e as práticas de literacia familiar realizadas com os pré-escolares.

### Análise e apresentação dos resultados

Os dados foram apresentados no formato de figuras e tabelas.

## RESULTADOS

### Seleção dos estudos primários

A estratégia de busca nas bases de dados recuperou 1092 referências, totalizando 1075 após a remoção dos duplicados. Durante a fase de leitura dos títulos e resumos, 1032 foram excluídas por não estarem nos critérios de elegibilidade. Foram selecionados 43 estudos para leitura na íntegra. Após a leitura, 14 estudos foram excluídos, resultando em 29 estudos incluídos para a síntese final da revisão de escopo (Figura 1).

**Figura 1:** Identificação dos estudos

### Características dos estudos

Os estudos incluídos foram publicados entre 2001 e 2023 em diversas revistas. Todos os artigos foram publicados em inglês e conduzidos em diferentes países, incluindo Estados Unidos (75,86%)^(16-37)^ Eslovênia (3,44%)^(38)^, Chile (3,44%)^(39)^, Grécia (3,44%)^(40)^, Austrália (10,34%)^(41-43)^ e China (3,44%)^(44)^ (Figura 1).

A idade dos participantes variou entre 9 meses e 5 anos e 11 meses de idade. Todos os estudos incluíram ambos os sexos. Os tamanhos das amostras variaram de 20 a 11.173 participantes. Em relação aos tipos de estudo, apenas foram selecionados estudos observacionais e experimentais.

Vinte e nove estudos citaram as práticas de literacia familiar; desses, 16 (55,17%) descreveram práticas formais e informais, 11 (37,96%) citaram práticas informais e 2 (6,8%) relataram apenas práticas formais.

Em relação às práticas de literacia familiar, as atividades informais apareceram com mais frequência. Entre os estudos, a prática informal de leitura de livros foi descrita em 20 (68,96%) estudos, seguida da leitura compartilhada citada em 10 (34,48%) estudos e o ensino da escrita mencionada em 10 (34,48%) pesquisas.

Quanto às práticas informais, 3 (10,34%) estudos citaram como atividades a contação de histórias, 4 (13,79%) artigos mencionaram cantar músicas, 2 (6,89%) estudos citaram brincar, 10 (34,48%) citaram a leitura compartilhada, 20 (68,96%) referiram ler para as crianças, 2 (6,88%) relataram utilizar jogos que contem letras e números e conversar com a criança sobre como foi o dia, 7 (24,13%) citaram colorir/desenhar, ler antes de dormir, ensinar cores e formas e ir à biblioteca^(16,17,19-21,27,30,33,35,38,43)^ (Tabela 1).

**Tabela 1:** Resumo das características descritivas gerais dos estudos incluídos (n=29)

**Autor**	**Participantes**	**Tipo de estudo**	**Métodos**	**Objetivos**	**Práticas de literacia familiar**
Haney e Hill^(43)^	Pré-escolares e seus pais (n-47).	Estudo transversal, quantitativo e qualitativo.	Foi realizada a aplicação do *Question from family literacy survey* com os pais dos pré-escolares. As crianças foram avaliadas com os instrumentos *Kaufman Survey of Early Academic and Language Skills (K-SEALS)* e Test of Early Reading Ability—Third Edition (TERA-3).	Analisar como o ambiente familiar impacta a alfabetização emergente.	Práticas formais: Nomes e sons das letras, escrever palavras, ler palavras ou ler livros. Práticas informais: não citado
Boudreau^(23)^	Pré-escolares e seus pais (n-37).	Estudo transversal quantitativo e qualitativo.	Foi realizada a aplicação do *Early Literacy Parent Questionnaire* com os pais dos pré-escolares, composto por 31 ítens. Além disso, as famílias responderam perguntas abertas sobre o ambiente de literacia.. As crianças foram avaliadas quanto às habilidades de rima, conhecimento de elementos impressos ao redor, como placas, marcas, nome e som das letras e narrativa.	Analisar a relação do conhecimento dos pais sobre alfabetização e o desempenho das crianças nas avaliações de habilidades iniciais.	Práticas formais: Produção de rimas, escrever, ensinar nomes de letras, sons de letras, consciência fonológica e alfabeto. Práticas informais: habilidades narrativas e interações com livros.
Jay e Rohl^(41)^	Pais de pré-escolares (n-9).	Estudo experimental longitudinal e quantitativo.	Foi realizada uma entrevista com os pais para investigar o ambiente de literacia familiar. Em seguida, as famílias participaram de um programa de intervenção (oficinas) com temas relacionados ao desenvolvimento da leitura e escrita. Por fim, realizaram uma autoavaliação sobre as mudanças no ambiente pós intervenção.	Analisar um programa de intervenção para pais de conscientização sobre o ambiente de alfabetização.	Práticas informais: Ler livros na hora de dormir, ouvi histórias gravadas, ida à biblioteca local, contação de histórias, brincar com o quebra-cabeça do alfabeto, criação de histórias para crianças, leitura de jornais, assisti à programas infantil, ir ao cinema e ao zoológico, piquenique juntos. Práticas formais: tarefas de leitura como lição de casa, ler livros da escola, conversar sobre alfabetização, atividades relacionadas a alfabetização.
Kim^(24)^	Pré-escolares e seus pais (n-215).	Estudo longitudinal.	Foi realizada aplicação do questionário sobre as práticas de literacia familiar com os pais. As crianças foram avaliadas quanto vocabulário, conhecimento do nome das letras, consciência fonológica, leitura de palavras, leitura de pseudopalavras, e soletração.	Investigar a relação das práticas de literacia e habilidades de alfabetização emergentes (vocabulário e consciência fonológica) e habilidades de alfabetização convencionais (leitura de palavras, leitura de pseudopalavras e ortografia).	Práticas formais: Ensinar o alfabeto e ajudar na lição de casa. Práticas informais: ter livros infantis; leitura compartilhada, leitura para criança, visita à biblioteca ou à livraria.
Hammer et al.^(29)^	Pré-escolares e suas mães (n-81).	Estudo transversal, descritivo.	Foi aplicado o questionário *Parental Modernity Scale e Rank Order of Parental Values,* com as mães das crianças que investigaram as crenças, valores e práticas utilizadas em casa.	Analisar o desenvolvimento da linguagem e da alfabetização de crianças bilíngues.	Práticas formais: escrever cartas, o alfabeto, os sons de letras, a contar, ensinar os números, ensinar a escrever, ensinar as formas. Práticas informais: Ler livro, ensinar as cores, colorir com a criança, ler a Bíblia, ler o jornal, ler a revista, ler boletins informativos da igreja, pegar um livro da biblioteca, fazer lista de compras,
Li et al.^(37)^	Pré-escolares (n-160)	Estudo longitudinal.	Foi aplicado com as crianças a *Preschool and Primary Chinese Literacy Scale* no início do estudo e após três anos. Os pais responderam ao questionário estruturado sobre frequência e tipo de práticas de literacia.	Investigar os efeitos das atividades formais de alfabetização nos resultados posteriores de alfabetização das crianças.	Práticas informais: Leitura de livros e leitura compartilhada. Práticas formais: ensinar a escrever.
Perry et al.^(28)^	Pré-escolares e suas mães (n-13)	Estudo longitudinal de intervenção.	Foi aplicado um questionário estruturado com os pais que verificou atividades de leitura e escrita realizadas em casa, uso de materiais de leitura, crenças dos pais sobre a literacia, envolvimento dos pais na educação das crianças. O questionário foi aplicado no início do estudo e após dois anos.	Examinar como as famílias imigrantes latinas incorporam atividades de alfabetização interativas baseadas na escola em suas casas.	Práticas informais: Jogos de alfabetização e leitura de livros. Práticas formais: ensino das cores, ensino das partes do corpo, associar a palavra escrita e a linguagem oral, ler palavras escritas em livros ou em jogos em voz alta.
Hood et al.^(42)^	Pré-escolares (n-143).	Estudo longitudinal.	Foi aplicado com os pais o *Home Literacy Environment Questionnaire* que avaliou práticas de leitura compartilhada entre pais e filhos e com as crianças o *Children’s Title Recognition Test.*	Verificar as práticas realizadas no ambiente familiar e sua relação com a alfabetização emergente.	Práticas formais: Ensinar o alfabeto, ensinar escrever o nome, ensinar a leitura. Práticas informais: não citado.
Burgess^(22)^	Pré-escolares e seus pais (n-262).	Estudo transversal.	Foi aplicado com os pais um questionário elaborado pelo pesquisador com perguntas sobre o ambiente de literacia familiar.	Compreender o ambiente de literacia familiar.	Práticas informais: Leitura de livros infantis, leitura de letras magnéticas, jogos de rima.
Hindman e Morrison^(21)^	Pré-escolares e seus pais (n-229).	Estudo transversal.	Pais e crianças foram filmados / observados enquanto realizavam uma atividade de escrita conjunta: escrever um convite para uma festa de aniversário. A tarefa ocorria numa visita domiciliar de 1 hora. Os pais preencheram um questionário sobre práticas de literacia.	Analisar o desenvolvimento da alfabetização e as práticas de literacia realizadas durante a primavera.	Práticas formais: Ensinar sons das letras, ensinar os nomes das letras, ensinar a ler palavras, incentivar a criança a escrever, fazer atividades relacionadas à matemática, Práticas informais: jogos de números com crianças, apoio à autonomia, encorajar a criança a explorar e questionar as coisas, passar tempo com a criança.
Skwarchuk et al.^(16)^	Pré-escolares e seus pais (n-279).	Estudo transversal.	Foi aplicado questionário com os pais sobre literacia e numeracia. As crianças responderam a testes que avaliavam conhecimento numérico e de leitura.	Examinar as expectativas acadêmicas, a alfabetização e atitudes de numeramento e práticas de literacia dos pais antes de seus filhos começarem a escola.	Práticas formais: Ler palavras, apontar palavras/letras, quando lemos durante a leitura, reconhecer letras escritas, escrita de palavras, identificação de palavras por meio de sinais (pare, siga), ensino dos sons das letras, apresentar palavras e os significados, Práticas informais: cantar músicas que envolvem o alfabeto, incentivo às rimas, realizar perguntas após leitura compartilhada, visita à biblioteca.
Förster e Rojas-Barahona^(39)^	Pré-escolares e seus pais (n-240).	Estudo transversal.	Foi aplicado com os pais um questionário sobre aspectos socioeconômicos e de literacia familiar. As crianças foram avaliadas por meio do Assessment Battery for Children K-ABC.	Analisar o impacto das práticas de alfabetização, variáveis ​​sociodemográficas e frequência a um programa de educação formal antes do desenvolvimento de habilidades de alfabetização.	Práticas informais: Ler, cantar músicas, contar histórias ou conversar sobre histórias, jogos com letras e números, jogos de palavras, ler sinais ou avisos, ler revista, falar sobre coisas que aconteceram durante o dia, falar sobre programas de televisão. Práticas formais: escrever cartas e palavras.
Schick e Melzi^(30)^	Pré-escolares e seus pais (n-127).	Estudo longitudinal.	Foi aplicado com as famílias um questionário sobre a frequência com a qual os cuidadores compartilhavam livros com as crianças, além da frequência de materiais impressos em casa. As crianças foram avaliadas quanto às habilidades de linguagem.	Analisar as práticas de literacia familiar no início do ano letivo e os resultados de linguagem, alfabetização e prontidão escolar socioemocional das crianças no final do ano pré-escolar.	Práticas informais: Leitura de livros, leitura compartilhada, elaboração de narrativa do cuidador, diálogo com a criança, apontar palavras nos rótulos dos alimentos e placas nas ruas, conversar sobre receitas enquanto cozinham. Práticas formais: pedir às crianças que escrevam seu nome ou outras palavras.
Sawyer et al.^(31)^	Pré-escolares e suas mães (n-20).	Estudo transversal.	Foi aplicado uma entrevista semi-estruturada com as mães sobre as práticas de literacia doméstica, crenças sobre como as crianças aprendem a ler, escrever linguagem, e fatores que influenciam essas práticas e crenças.	Analisar o ambiente de práticas de literacia familiar, quais crenças as mães têm sobre como as crianças aprendem a ler e quais fatores influenciaram as crenças e práticas das mães.	Práticas informais: Ir à biblioteca, ler livros, colorir/desenhar, envolvimento com livros. Práticas formais: Não citou.
Bingham et al.^(20)^	Pré-escolares e seus pais (n-181).	Estudo exploratório.	Foi aplicado com os pais questionários de auto-relato para indicar até que ponto concordam com afirmações representativas de estilos parentais permissivos e autoritários e um formulário de literacia familiar divididas em atividades formais e informais. Foi realizada avaliação da linguagem oral das crianças com os seguintes testes: *Peabody Picture Vocabulary Test – Fourth Edition (PPVT-4)* e *Test of Preschool Early Literacy (TOPEL).*	Analisar estilos parentais e práticas de alfabetização e as correlações de habilidades de linguagem oral.	Práticas informais: Leitura compartilhada de livros, leitura de livros, ida à biblioteca, leitura de livro ilustrado.
Gonzalez et al.^(32)^	Pré-escolares e seus pais (n-252).	Estudo clínico randomizado.	Foi aplicado questionário com as mães sobre o contexto socioeconômico, educação materna, ambiente de literacia, crenças de leitura maternas e frequência de leitura compartilhada. As crianças foram avaliadas quanto ao vocabulário.	Analisar se as características sociodemográficas dos pais estão associadas às crenças e atitudes que os pais têm sobre o desenvolvimento da linguagem e da alfabetização de seus filhos.	Práticas informais: Leitura de livros.
van Bysterveldt e Westerveld^(45)^	Pré-escolares e seus pais (n-77).	Estudo transversal.	Foram aplicados dois questionários: sociodemográfico e Zarit Burden Interview (versão de 12 itens).	Analisar habilidades emergentes de alfabetização e cognição.	Práticas informais: Ler livros, ter livros em casa, leitura compartilhada, jogos de rima. Práticas formais: ler palavras, nome das letras, escrever palavras, nomear palavras.
Marjanovič-Umek et al.^(38)^	Pré-escolares e suas mães (n-20).	Estudo observacional.	Foi utilizado *Scale for Observing Shared Reading* para verificar a qualidade da leitura compartilhada, *Frog Goes to Dinner* para avaliar a narrativa da criança e um questionário sobre o ambiente de literacia familiar.	Analisar as relações entre a qualidade da leitura compartilhada mãe-filho, a habilidade de contar histórias da criança e o ambiente de alfabetização em casa.	Práticas informais: Imita a voz da criança ou onomatopeias, ler livros, leitura compartilhada, conversa com a criança sobre as ilustrações, responde às perguntas da criança, elogia a criança; Conecta o conteúdo da história à vida cotidiana e às experiências reais da criança; Descreve sentimentos e desejos; Descreve estados mentais; Usa metáforas; Faz perguntas sobre relações de causa e efeito; Faz perguntas que incluem processos metacognitivos; Atenção conjunta com uma criança. Práticas formais: explica as características das palavras, solicita que a criança explique o significado das palavras.
Puranik et al.^(18)^	Pré-escolares e seus pais (n-151).	Estudo transversal.	Foi aplicado questionário sobre as práticas de literacia familiar com os pais e realizado avaliação da escrita com as crianças.	Analisar quais os tipos de atividades relacionadas à escrita os pais realizam com seus filhos em idade pré-escolar e se as práticas de alfabetização em casa contribuem para as habilidades iniciais de escrita das crianças.	Práticas formais: Ensinar letras, escrever cartas, escrever o nome, atividades de escrita juntos, escrita de bilhetes ou cartões de aniversário, escrever nomes/palavras.Práticas informais: não citou.
Roopnarine e Dede Yildirim^(19)^	Pré-escolares e seus pais (n-11.173)	Estudo transversal.	Foi aplicado um questionário com os pais sobre engajamento em atividades cognitivas, recursos de alfabetização no lar e habilidades de alfabetização das crianças.	Analisar o envolvimento de mães e pais em brincadeiras, leitura de livros e atividades de contar histórias.	Práticas informais: Brinquedos caseiros, brinquedos de lojas, ler livros, contar histórias e brincar. Práticas formais: identificar letras, ler palavras.
Riser et al.^(17)^	Pré-escolares e seus pais (n-10.400)	Estudo transversal.	Foi observado o envolvimento dos pais em atividades de literacia familiar e avaliado as crianças com relação às habilidades acadêmicas.	Análise secundária de dados incluindo entrevistas e questionários com os pais, registros de certidões de nascimento, visitas domiciliares, professores, entrevistas e questionários, observações em sala de aula e avaliações diretas das crianças.	Práticas informais: Leitura compartilhada, contação de histórias e cantar canções. Práticas formais: não citou.
Myrtil et al.^(25)^	Pré-escolares e seus pais (n-466)	Estudo transversal.	Foi aplicado com os pais um questionário sobre as práticas de literacia familiar e avaliado a linguagem oral das crianças.	Examinar a inter-relação do ambiente de alfabetização doméstica de famílias rurais de baixa renda (interações entre pais e filhos, interesse da criança, uso da biblioteca e acesso a livros) e determinar até que ponto as características do cuidador e da criança predizem essas dimensões.	Práticas informais: Ler livros, cantar e recitar rimas , contar histórias, fazer desenhos, ir à biblioteca. Práticas formais: escrever cartas.
Lin et al.^(33)^	Pré-escolares e seus pais (n-262).	Estudo correlacional.	Foram aplicados os questionários sobre práticas de literacia, comunicação entre pais e educadores e variáveis demográficas.	Examinar a relação entre as percepções dos pais sobre a comunicação entre pais e educadores e os ambientes de aprendizagem em casa das crianças.	Práticas informais: Ler livros contendo números, ler livros. Práticas formais: nomeação de letras escritas, identificar sons de letras.
Eutsler e Trotter^(35)^	Pré-escolares e seus pais (n-37)	Estudo trata-se de casos múltiplos.	Foi aplicado com os pais um questionário sobre as práticas de literacia. Sessões de leitura compartilhada (pais e criança), sendo duas sessões com textos impressos e duas com textos digitais. Observação comportamental das crianças.	Analisar as práticas de alfabetização doméstica das crianças e preferência textual das crianças para leitura em uma sala de aula dentro do ambiente escolar.	Práticas informais: Leitura de livro impresso ou digital, jogos de imaginação, dispositivos tecnológicos.
Chen et al.^(26)^	Pré-escolares e seus pais (n-4.907).	Estudo transversal.	Foi aplicado com os pais um questionário sobre as práticas de literacia e variáveis demográficas.	Investigar como diferentes dispositivos (incluindo TV, tablet, computador e livros em papel) podem canalizar a eficácia parental (ou a falta dela) para práticas de alfabetização doméstica.	Práticas informais: Leitura de livro e contação de histórias.
Tsirmpa et al.^(40)^	Pré-escolares e seus pais (n-147).	Estudo transversal.	Os pais responderam a entrevistas semi-estruturadas sobre as crenças e práticas de literacia familiar.	Analisar as crenças sobre alfabetização e o desenvolvimento da alfabetização.	Práticas informais: Ler livros, nomear cores. Práticas formais: apontar letras ou números.
Dulin et al.^(27)^	Pré-escolares e suas mães (n-13)	Estudo longitudinal	Foi aplicado com os pais um questionário sobre as práticas de literacia. As crianças foram avaliadas por meio do teste MacArthur-Bates.	Caracterizar os ambientes de alfabetização domiciliar de crianças com síndrome de Down e examinar se a riqueza do ambiente de alfabetização, o envolvimento da criança durante atividades de leitura compartilhada de livros de histórias, a qualidade da atividade de leitura compartilhada de livros de histórias entre cuidador e criança e a exposição à linguagem no ambiente doméstico previram o vocabulário receptivo da criança simultaneamente.	Práticas informais: Leitura compartilhada, leitura de livros, narração de histórias, nomeação de imagens em livros. Práticas formais: Não citou.
DesJardin et al.^(36)^	Pré-escolares e seus pais (n-36).	Estudo transversal.	Foi aplicado com os pais um questionário sobre as práticas de literacia. As crianças foram avaliadas com relação à linguagem oral e interações de leitura compartilhada.	Investigar a quantidade (por exemplo, frequência de leitura compartilhada, prazer da criança) e a qualidade (comportamentos dos pais e das crianças) da leitura compartilhada de livros em crianças em idade pré-escolar com e sem perda auditiva.	Prática informal: Leitura compartilhada. Prática formal: não citou.

As práticas, a citar: conversar com a criança sobre as ilustrações do livro, responder as perguntas da criança, elogiar, conectar conteúdo da leitura às atividades de vida diária, fabricar brinquedos, assistir juntos, brincar com quebra-cabeças, ir ao cinema, ir ao zoológico, fazer piquenique, ler a bíblia revistas e jornais foram citadas em 1 (3,44%) estudo^(16,17,19-21,27,30,33,35,38,43)^ (Tabela 1).

Se tratando das práticas formais, 10 (34,48%) estudos citaram escrita de palavras, 2 (6,88%) citaram ensinar a ler, ensinar o alfabeto, realizar atividades da escola, ensinar o nome das letras, 5 (17,20%) estudos citaram a produção de rimas, 1 (3,44%) estudo referiu ensinar escrever o próprio nome e identificação de grafema e fonema^(16,18,21,23,42)^, (Tabela 1).

As práticas de literacia citadas nos estudos aqui descritos foram realizadas pelos pais, com predomínio da figura materna na realização das atividades formais e informais.

## DISCUSSÃO

Esta revisão de escopo compilou os dados a respeito das práticas de literacia familiar realizadas com os pré-escolares, classificadas nos estudos aqui descritos enquanto formais e informais. As práticas formais estão relacionadas ao ensino do nome das letras, ensino dos sons das letras, leitura de palavras e escrita. Essas atividades se associam ao reconhecimento do alfabeto no período pré-escolar e promovem o desenvolvimento de habilidades preditoras para linguagem escrita^(16,17)^.

As práticas informais se referem às atividades que estimulam o desenvolvimento das crianças e posteriormente, estarão associadas às habilidades necessárias para aprendizagem formal da leitura e escrita. Nos estudos revisados foram descritas as práticas de leitura compartilhada, contação de histórias, idas à biblioteca, cantar, fabricar os próprios brinquedos, desenhar e assistir juntos^(16,20,21,23,38)^.

Há relação entre práticas informais e habilidades de linguagem oral, a exemplo dos estudos de Dulin et al.^(27)^, Bingham et al.^(20)^, Puranik et al.^(18)^, Marjanovič-Umek et al.^(38)^, Skwarchuk et al.^(16)^, Hindman e Morrison^(21)^ e Kim^(24)^ que investigaram as práticas informais e seus impactos na linguagem^(16,17,20,21,27)^. Os artigos apresentaram práticas como, leitura de livros, ofertar livros para as crianças, leitura compartilhada e atividades com músicas, e relacionou essas atividades a melhores desempenhos nas avaliações de cognição, bem como, impactos a nível de vocabulário, consciência fonológica e leitura de palavras^(16,20,21,38)^.

No estudo de Riser et al.^(17)^, realizado com 10.400 pré-escolares descendentes de tribos indígenas, a associação das práticas informais à resultados positivos para escala de cognição foi enfatizada, mas, sob a perspectiva de atividades relacionadas à própria cultura, por meio de contação de histórias e de canções. Além disso, havia uma associação entre as práticas de literacia, a renda e o nível de escolaridade materna.

As práticas informais também foram descritas no estudo de Roopnarine e Dede Yildirim^(19)^ que objetivou comparar a participação materna e paterna no ambiente familiar. Dentre os cinco países pesquisados neste estudo, apenas um país teve participação dos pais mais ativa na realização de brincadeiras livres, contação de histórias e fabricação de brinquedos. Nos demais países, as mães eram muito mais propensas a se envolver nas práticas, ademais, as contações de histórias e brincadeiras com as mães foram significativamente e positivamente associadas à identificação de letras^(19)^.

O envolvimento das mães nas práticas de literacia e suas repercussões no desenvolvimento das crianças foi explorado no estudo de Bingham et al.^(20)^, que verificou a qualidade da leitura compartilhada entre mãe-filho, além disso, nos estudos de Hammer et al.^(29)^, Tsirmpa et al.^(40)^, Burgess^(22)^ e Dulin et al.^(27)^, observa-se maior envolvimento da figura materna na execução das atividades^(27,29,40)^. Isso pode estar associado à cultura do cuidado dos filhos à mãe, visto que, ao verificarmos estudos que retratam o cuidador principal, a figura materna é evidenciada de forma mais ativa nesse cuidado^(44,46,47)^.

A respeito das práticas formais, os estudos de Puranik et al.^(18)^, Skwarchuk et al.^(16)^, Hindman e Morrison^(21)^, Hood et al.^(42)^ e Kim^(24)^ observaram correlações entre atividades envolvendo a escrita e suas repercussões para a aprendizagem emergente da escrita, bem como os impactos na linguagem oral e habilidades de leitura^(23,42)^. Além disso, a pesquisa de Puranik et al.^(18)^ enfatizou as correlações entre a escolaridade dos pais e as habilidades de escrita de cartas, ortografia e escrita espontânea das crianças.

No estudo de Kim^(24)^ em que verificou práticas formais, práticas informais e as repercussões no aprendizado de leitura de palavras e pseudopalavras, observou que as crianças expostas à leitura apresentavam desempenho mais elevado na leitura quando comparado os pré-escolares que vivenciam atividades formais, como ensino do nome das letras.

Na pesquisa de Perry et al.^(28)^ que objetivou verificar as preferências entre atividades formais ou informais, os pais relataram preferir as práticas informais voltadas para brincadeiras, envolvendo jogos de tabuleiro, desenhos e leitura de livros de forma lúdica com variações nas entonações vocais.

Apesar da preferência pelas atividades informais, os pais relataram apoiar o desenvolvimento de conhecimento numérico e de vocabulário de seus filhos por meio de atividades como contagem repetitiva e prática de associações de letras e sons^(28)^. Ademais, os pais identificaram atividades de alfabetização como uma ferramenta para transmitir uma mensagem moral ou orientar as crianças para um bom comportamento^(28)^.

Na pesquisa de Tsirmpa et al.^(40)^ foi observado as crenças dos pais referentes aos tipos de práticas que deveriam ser realizadas com os pré-escolares. Observou-se que os pais mais convencionais acreditavam que as crianças devem estar familiarizadas com as letras e números e ser capazes de reconhecer e escrever alguns deles. Em contrapartida, os pais adeptos da educação facilitadora acreditavam que as habilidades motoras finas, percepção visual e coordenação olho-mão deveriam ser trabalhadas, bem como a linguagem oral, para promover a aprendizagem da linguagem escrita^(40)^.

Com relação à escolaridade, foi observado nos estudos de Riser et al.^(17)^, Puranik et al.^(18)^ e Skwarchuk et al.^(16)^ associação entre as práticas de literacia, melhores desempenho nas avaliações de linguagem e nível educacional dos pais^(22,40)^. Ademais, foi verificado que os pais com nível educacional mais elevado consideravam a família como fator primordial no processo de ensino-aprendizagem dos pré-escolares, por outro lado, os pais com nível educacional menos elevado, acreditavam que a escola é a principal responsável por ensinar as crianças^(40)^.

Em contrapartida aos estudos acima citados, nas pesquisas de Förster e Rojas-Barahona^(39)^ e Burgess^(22)^ não houve diferença entre os níveis de escolaridade e as atividades envolvendo leitura. Por outro lado, ao analisarmos a renda socioeconômica, esta estava associada à mais oferta de recursos como livros, jogos com letras e brinquedos^(25,26)^.

No mais, todos os estudos incluídos na revisão citaram a prática informal de leitura de livros, e todos os estudos citaram os impactos das práticas de literacia familiar no desenvolvimento das crianças. Quando comparamos com achados presentes na literatura, a participação da família é enfatizado em diversos estudos como sendo primordial para o sucesso educacional, desenvolvimento linguístico e social, dessa forma, esses dados estão em concordância^(48,49)^.

Nos artigos incluídos foi possível observar que dentre os 29 estudos, 27 foram realizados por países desenvolvidos^(16-37)^. Esse dado pode ter relação com uma maior preocupação desses países acerca do desenvolvimento das crianças e da escolarização, visto que, crianças fora da escola ou que abandonam a escola estão entre os problemas sociais mais importantes do mundo, especialmente em países de baixa e média renda.

De acordo com o Artigo 26 da Declaração Universal dos Direitos Humanos, os países precisam fornecer a todos o acesso à educação gratuita, além de investir recursos significativos em instalações educacionais, contudo, ao realizar esta revisão, e observar que apenas uma pesquisa foi realizada em um país em desenvolvimento, isso pode ter relação com a evasão escolar citada no estudo acima, bem como, ter associação com os índices educacionais nos países em desenvolvimento.

Dessa forma, a continuidade de pesquisas que visem explorar os aspectos da literacia familiar é primordial. Além disso, realizar estudos experimentais, que observem os resultados das atividades de literacia com famílias em vulnerabilidade social podem contribuir com a educação e por conseguinte, diminuir a evasão escolar, e melhorar os índices educacionais em países em desenvolvimento.

No que se refere às limitações do presente estudo é possível destacar aspectos metodológicos dos artigos encontrados, a citar: ausência de grupo controle nos estudos de intervenção e a distribuição aleatória dos participantes. Quanto aos estudos observacionais, o uso de entrevistas semi-estruturadas com perguntas abertas para investigar os tipos de práticas, pode ter comprometido os resultados se comparado aos que utilizaram protocolos validados.

## CONCLUSÃO

A partir do mapeamento das práticas realizadas com os pré-escolares, foi possível observar o predomínio das práticas informais. Dentre as atividades realizadas, a mais citada foi a leitura de livros e, em seguida, a leitura compartilhada. No mais, brincar juntos, fabricar os próprios brinquedos, desenhar, colorir, cantar, ouvir músicas e realizar passeios, foram atividades relatadas pelos cuidadores. Se tratando das práticas formais, ensinar a escrever foi a prática mais descrita.Por fim, há um consenso sobre a importância do ambiente familiar na aprendizagem das crianças e a relação das práticas de literacia familiar com o desenvolvimento da linguagem oral e posteriormente linguagem escrita.

## Supplementary Material

Supplementary material accompanies this paper.

Legenda S1 Appendix A. Database Search Strategy

Legenda S2 Appendix B. Excluded Articles

This material is available as part of the online article from https://DOI: 10.1590/2317-1782/e20250148en
